# Crystal structure of the N-terminal domain of MinC dimerized *via* domain swapping

**DOI:** 10.1107/S0909049513022760

**Published:** 2013-10-02

**Authors:** Jun Yop An, Tae Gyun Kim, Kyoung Ryoung Park, Jung-Gyu Lee, Hyung-Seop Youn, Youngjin Lee, Jung Youn Kang, Gil Bu Kang, Soo Hyun Eom

**Affiliations:** a School of Life Sciences, Gwangju Institute of Science and Technology, Gwangju 500-712, Republic of Korea; b Steitz Center for Structural Biology, Gwangju Institute of Science and Technology, Gwangju 500-712, Republic of Korea

**Keywords:** MinC, crystal structure, domain swapping, FtsZ ring, cell division

## Abstract

Proper cell division at the mid-site of gram-negative bacteria reflects critical regulation by the *min* system (MinC, MinD and MinE) of the cytokinetic Z ring, which is a polymer composed of FtsZ subunits. MinC and MinD act together to inhibit aberrantly positioned Z-ring formation. MinC consists of two domains: an N-terminal domain (MinC_NTD_), which interacts with FtsZ and inhibits FtsZ polymerization, and a C-terminal domain (MinC_CTD_), which interacts with MinD and inhibits the bundling of FtsZ filaments. These two domains reportedly function together, and both are essential for normal cell division. The full-length dimeric structure of MinC from *Thermotoga maritima* has been reported, and shows that MinC dimerization occurs *via* MinC_CTD_; MinC_NTD_ is not involved in dimerization. Here the crystal structure of *Escherichia coli* MinC_NTD_ (*Eco*MinC_NTD_) is reported. *Eco*MinC_NTD_ forms a dimer *via* domain swapping between the first β strands in each subunit. It is therefore suggested that the dimerization of full-length *Eco*MinC occurs *via* both MinC_CTD_ and MinC_NTD_, and that the dimerized *Eco*MinC_NTD_ likely plays an important role in inhibiting aberrant Z-ring localization.

## Abstract 2

The crystal structure of *Eco*MinC_NTD_ dimerized *via* domain swapping was solved at 2.3 Å resolution. The present study suggests that *Eco*MinC dimerizes through both *Eco*MinC_NTD_ and *Eco*MinC_CTD_.

## 1. Introduction

Cytokinesis in bacteria is carried out by the cytokinetic ring (FtsZ ring or Z ring), which acts in part by recruiting other cell-division proteins (Lutkenhaus, 1998 ▶, 2007 ▶; Dajkovic & Lutkenhaus, 2006 ▶). The Z ring, which is a polymer composed of FtsZ subunits, is normally situated at the mid-site of cells undergoing division, but it associates with the membrane through ZipA and FtsA, and in the absence of the *min* system (MinC, MinD and MinE) can be moved from the mid-site to the polar regions of cells (Yu & Margolin, 1999 ▶). For successful cell division in *Escherichia coli*, cooperative behavior among the Min proteins is required (de Boer *et al.*, 1989 ▶; Rothfield *et al.*, 1999 ▶). MinC is a critical regulator of FtsZ polymerization that is bound to MinD and oscillates from one polar region within the cell to the other. By destabilizing FtsZ polymers in the polar regions, MinC acts to inhibit the division process in those regions (Hu & Lutkenhaus, 1999 ▶; Raskin & de Boer, 1999*a* ▶). MinD, which attaches to the membrane through a C-terminal amphipathic helix that embeds into membrane bilayer following ATP-dependent dimerization, recruits MinC to the membrane (Szeto *et al.*, 2003 ▶; Hu & Lutkenhaus, 2003 ▶; Zhou & Lutkenhaus, 2003 ▶; Hu *et al.*, 2003 ▶; Lackner *et al.*, 2003 ▶). In that way, MinD can increase the MinC concentration at the membrane by up to 50-fold (de Boer *et al.,* 1992 ▶; Hu *et al.*, 1999 ▶; Raskin & de Boer, 1999*b* ▶). The MinC/D complex is regulated by MinE, which restricts localization of the complex to the polar regions, thereby limiting assembly of FtsZ polymers to the mid-site (de Boer *et al.*, 1989 ▶). MinE accomplishes this regulation by stimulating the ATPase activity of MinD and dissociating MinD from the membrane (Hu & Lutkenhaus, 1999 ▶, 2001 ▶; Raskin & de Boer, 1999*a* ▶,*b* ▶; Fu *et al.*, 2001 ▶; Hale *et al.*, 2001 ▶; Hu *et al.*, 2002 ▶; Shih *et al.*, 2003 ▶). Through this cooperative behavior among Min proteins, the Z ring is stably located at the mid-site, enabling division of a cell into two daughter cells to occur normally.

MinC is composed of two domains. Its N-terminal domain (MinC_NTD_) interacts with α10 of FtsZ, weakening the longitudinal bonds between FtsZ subunits within filaments, which leads to a loss of polymer rigidity and polymer shortening (Dajkovic *et al.*, 2008 ▶). On the other hand, the C-terminal domain (MinC_CTD_) interacts with MinD and the C-terminus of FtsZ to inhibit the bundling of FtsZ filaments. (Hu & Lutkenhaus, 2000 ▶; Dajkovic *et al.*, 2008 ▶; Shen & Lutkenhaus, 2009 ▶). It has been proposed, however, that at physiological levels the most likely function of the interaction between MinC_CTD_ and the C-terminal tail of FtsZ is to target MinC_NTD_ to FtsZ polymers. Thus, the mechanism of Z-ring inhibition by MinC may involve two simultaneous interactions of MinC with FtsZ: MinC_NTD_ binding to α10 of FtsZ that is important for polymer assembly, and MinC_CTD_-mediated targeting of MinC to FtsZ (Blasios *et al.*, 2013 ▶).

The structure of MinC has been reported for the hyperthermophilic bacterium *Thermotoga maritima* (*Tma*MinC) [Cordell *et al.*, 2001 ▶; Protein Data Bank (PDB) ID 1hf2]. Dimerization of *Tma*MinC is mediated solely by the MinC_CTD_ domain; MinC_NTD_ is not involved in dimerization in this species. By contrast, the crystal structure of MinC_NTD_ from *Salmonella typhimurium* (*Sty*MinC_NTD_) was found to be dimeric (PDB ID 3ghf, unpublished). Thus the mode of MinC dimerization and the mechanism by which the dimer inhibits FtsZ assembly is not yet fully understood. We targeted *E. coli* MinC (*Eco*MinC) for a structural study and determined the crystal structure of the dimeric *Eco*MinC_NTD_ at 2.3 Å resolution. *Eco*MinC_NTD_ forms a dimer *via* domain swapping between the first β strands in each subunit, as observed in the *Sty*MinC_NTD_ structure. Moreover, we found that dimerization of full-length *Eco*MinC is mediated not only by MinC_CTD_ but also by MinC_NTD_. We suggest that dimerized *Eco*MinC_NTD_ plays a key role in the inhibition of aberrant FtsZ polymerization.

## 2. Materials and methods

### 2.1. Cloning, expression and purification

The recombinant *Eco*MinC_NTD_ gene (residues 1–105) was amplified from *E. coli* (ATCC No. 700926D-5) genomic DNA using PCR, and restriction enzyme sites were added using gene-specific primer pairs. The PCR product was recombined into the modified pET-28a vector using the *Bam*HI/*Xho*I site, after which the recombinant plasmid was transformed into *E. coli* strain BL21 (DE3) for overexpression of protein. The transformants were grown in Luria-Bertani (LB) medium containing 50 µg ml^−1^ kanamycin at 310 K to an OD_600_ of approximately 0.7, at which time 0.5 m*M* isopropyl β-d-1-thiogalactopyranoside (IPTG) was added to induce expression of the recombinant protein, and the cells were incubated for an additional 9 h at the same temperature. The cells were harvested by centrifugation at 4500 × *g* for 15 min at 277 K, resuspended with buffer A (50 m*M* sodium phosphate [pH 8.0], 300 m*M* NaCl and 5 m*M* imidazole), and lysed by sonication. The crude lysate was centrifuged at 16000 × *g* for 50 min at 277 K, and the supernatant was loaded onto a Ni-NTA column (Peptron) previously equilibrated with buffer A. The protein was eluted with buffer B (50 m*M* sodium phosphate [pH 8.0], 300 m*M* NaCl and 300 m*M* imidazole). The eluate was concentrated using a Centriprep YM-3 (Millipore) and incubated with TEV protease at 277 K overnight to remove the hexahistidine tag. The protein was then further purified by size-exclusion chromatography using a Superdex 200 16/60 column (GE Healthcare, USA) equilibrated with buffer C (20 m*M* Tris-HCl [pH 8.0], 150 m*M* NaCl and 1 m*M* DTT). Finally, the eluate was concentrated to 14 mg ml^−1^ using a Centriprep YM-3 (Millipore) for crystallization. The protein concentration was determined spectrophotometrically using an extinction coefficient of 5504 *M* ^−1^ cm^−1^ (molecular weight = 11863 Da) at a wavelength of 280 nm.

### 2.2. Crystallization and X-ray analysis

Initial crystallization conditions were found in a Crystal Screen I and II reagent kit (Hampton Research) using Intelliplate crystallization trays with 80 µl of well solution and 1.0 µl drop (equal volume of protein and well solutions) in a sitting-drop 96-well format at 294 K. The crystallization conditions were then further refined using the hanging-drop vapor-diffusion method with a 2 µl drop. The best crystals were observed after three days in well solution consisting of 1.4 *M* sodium citrate (pH 6.5). For X-ray diffraction experiments, the crystals were flash frozen in liquid nitrogen using Paraton-N as a cryoprotectant. The diffraction dataset was collected on beamline 4A (MXW) at the Pohang Accelerator Laboratory (Pohang, South Korea) at a wavelength of 1.0000 Å using an ADSC Quantum 315 CCD detector. The data set was processed and scaled using *HKL2000* (Otwinowski & Minor, 1997 ▶).

### 2.3. Structure determination and refinement

The structure was determined at 2.3 Å resolution by molecular replacement using *PHENIX* (Adams *et al.*, 2010 ▶). Monomeric *Sty*MinC_NTD_ (PDB ID 3ghf) was used as a search model, and two molecules in an asymmetric unit were identified. The structures of *Eco*MinC_NTD_ were subjected to many cycles of manual rebuilding using the program *COOT* (Emsley *et al.*, 2010 ▶), and were refined through series of simulated annealing, rigid body, group *B*-factor, individual *B*-factor and TLS refinements using the program *PHENIX*. The final structure was obtained with *R*_work_ = 0.229 and *R*_free_ = 0.263. The statistics for the data collection and structure refinement are summarized in Table 1 ▶.

## 3. Results and discussion

### 3.1. Structure of *Eco*MinC_NTD_

**Figure 1:**
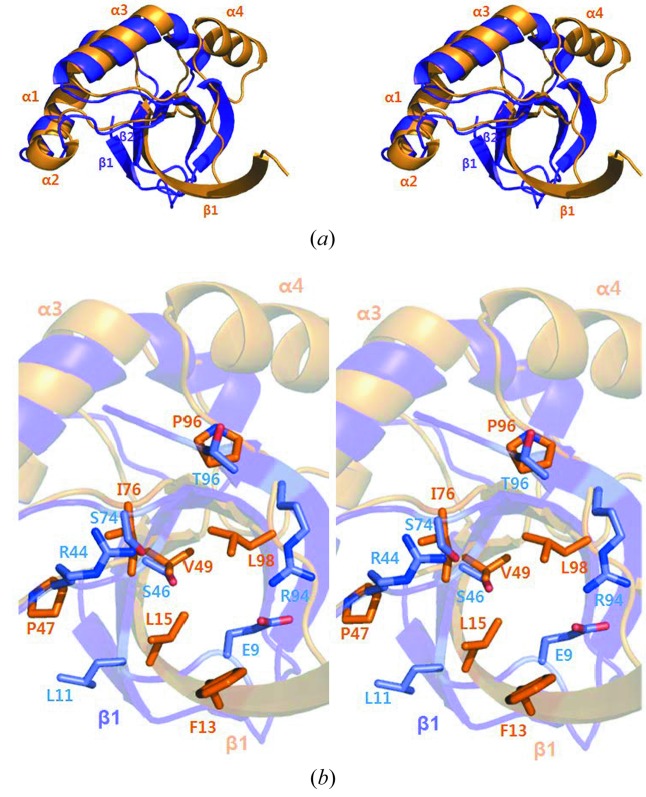
Overall structure of the monomeric MinC N-terminal domain from *Escherichia coli* (*Eco*MinC_NTD_). (*a*) Structural comparison of *Eco*MinC_NTD_ (orange) and the *T. maritima* MinC N-terminal domain (*Tma*MinC_NTD_, purple). Superimposition was performed using the program *lsqkab* in the CCP4i suite (Afonine *et al.*, 2005 ▶). (*b*) Comparison of residues in the central regions of *Eco*MinC_NTD_ (orange) and *Tma*MinC_NTD_ (purple).

The crystal structure of *Eco*MinC_NTD_ was solved at 2.3 Å resolution using the molecular replacement method. The search model was *Sty*MinC_NTD_ (PDB ID 3ghf), which has 84% sequence identity with the *E. coli* molecule. Like that of *Sty*MinC_NTD_, the architecture of monomeric *Eco*MinC_NTD_ includes three β strands and four α helixes (Fig. 1*a* ▶), and the two MinC_NTD_ structures superimposed with a root-mean-square deviation (RMSD) of 1.49 Å for the 97 Cα atoms (residues 5–101). By contrast, superimposition of the structures of *Eco*MinC_NTD_ and *Tma*MinC_NTD_ shows that whereas the first β strand of *Eco*MinC_NTD_ is unexpectedly long (residues 6–20), *Tma*MinC_NTD_ has two β strands forming an antiparallel β sheet in this region (β1 and β2 region; residues 3–6 and 11–15) (Fig. 1*a* ▶). In addition, residues in the central region of *Eco*MinC_NTD_ are mainly hydrophobic, while they are mainly polar in *Tma*MinC_NTD_ (Fig. 1*b* ▶).

**Figure 2:**
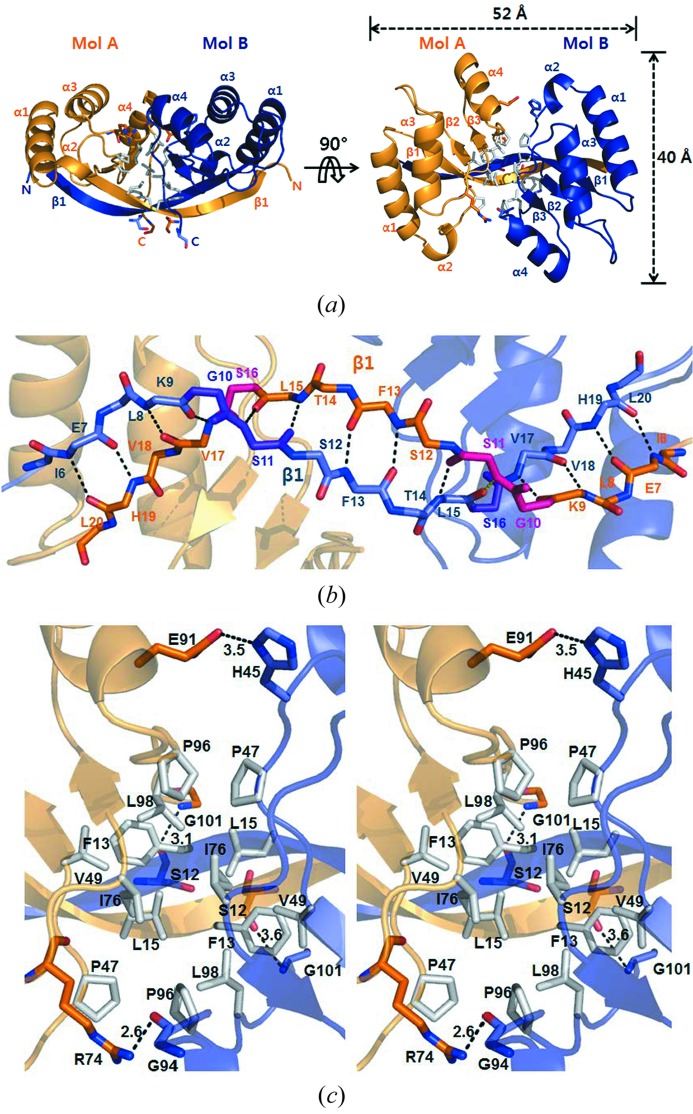
Interactions of dimeric *Eco*MinC_NTD_. (*a*) *Eco*MinC_NTD_ is dimerized through hydrogen bonds, hydrophobic interactions and antiparallel β1–β1 interactions between subunits. (*b*) Expanded view of the antiparallel β1–β1 interactions. (*c*) Expanded view of the central region. Hydrophobic residues are colored gray. Hydrogen bonds are shown as dashed lines.

**Figure 3:**
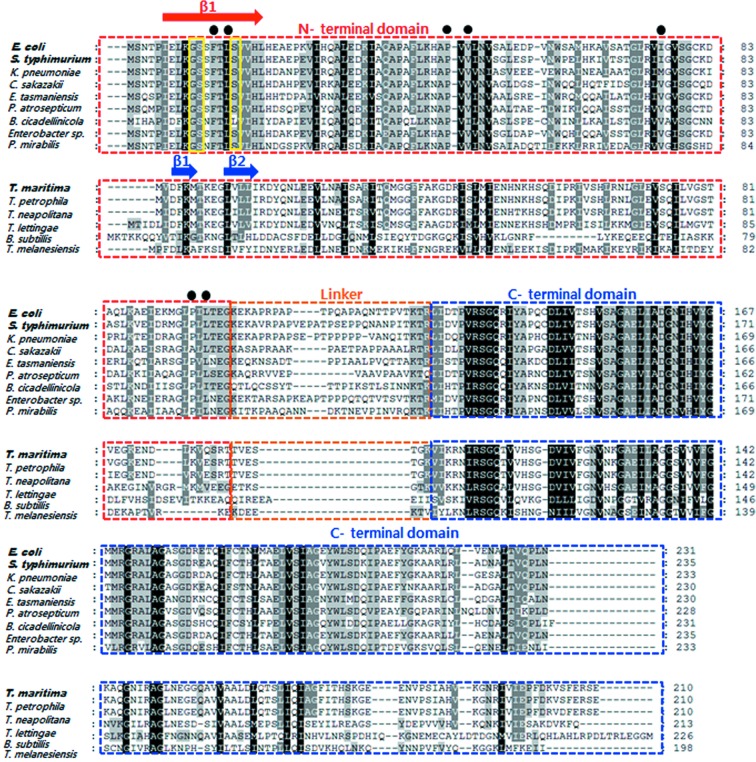
Multiple sequence alignment of MinC. Alignment was performed using the program *ClustalX* (Thompson *et al.*, 2002 ▶) and included MinC from *Escherichia coli*, *Salmonella typhimurium*, *Klebsiella pneumoniae* 342, *Cronobacter sakazakii* ATCC BAA-894, *Erwinia tasmaniensis*, *Pectobacterium atrosepticum* SCRI1043, *Baumannia cicadellinicola*, *Enterobacter sp.* 638 and *Proteus mirabilis* HI4320. A separate box was used for *Thermotoga maritima*, *Thermotoga petrophila* RKU-1, *Thermotoga neapolitana* DSM 4359, *Thermotoga lettingae* TMO, *Bacillus subtilis* and *Thermosipho melanesiensis* BI429. Residues involved in hydrophobic interactions in the central region of MinC_NTD_ are marked with black dots. Residues that are important for maintaining the long twisted antiparallel β1–β1 interaction are shown in yellow boxes. Each domain is boxed in a different color. The β1 in *Eco*MinC_NTD_ and β1–β2 strands in *Tma*MinC_NTD_ are shown. The species from which the MinC structure was determined is shown in bold.

We observed *Eco*MinC_NTD_ as a dimer within the asymmetric unit. The dimer is formed *via* domain swapping; that is, an antiparallel β1–β1 interaction between subunits [Figs. 2(*a*) and 2(*b*) ▶], as observed in the *Sty*MinC_NTD_ structure. Within the β1 strand, Gly10, Ser11 and Ser16 are important for mediating the long twisted antiparallel β1–β1 interaction (Fig. 2*b* ▶). In addition, the dimer is further stabilized by hydrogen bonds (Glu91–His45, Arg74–Gly94 and Ser12–Gly101) and hydrophobic interactions (among Phe13, Leu15, Pro47, Val49, Ile76, Pro96 and L98) at the central interface (Fig. 2*c* ▶). By contrast, *Tma*MinC lacks the corresponding Gly and Ser residues and hydrophobic residues that stabilize the domain swapped β1–β1 interaction. Instead, *Tma*MinC has an 8-KEG-10 sequence between two short β strands, which prevents formation of a long β strand (Fig. 3 ▶). Structural analysis of full-length *Tma*MinC has shown that it dimerizes through interaction of only *Tma*­MinC_CTD_ domains (Cordell *et al.*, 2001 ▶). There is no interaction between *Tma*MinC_NTD_ domains. To generate monomeric *Eco*MinC_NTD_, this antiparallel β1–β1 interaction should be broken and the hydrophobic residues will become exposed, because the long β1 strand cannot fold back to make the β-hairpin structure, as observed in the structure of *Tma*MinC_NTD_. Consequently, the shift from monomer to dimer is probably energetically stable in *Eco*MinC_NTD_. Thus we see dimeric *Eco*MinC_NTD_ in the result of size-exclusion chromatography, in a broad range of protein concentrations, as well as in the *Eco*MinC_NTD_ crystal structure.

Reportedly, unfused *Eco*MinC full-length and *Eco*MinC_CTD_ form dimers at a concentration of ≥10 µ*M*, which is consistent with our results (Szeto *et al.*, 2001 ▶). In the case of *Eco*MinC_NTD_, the MalE fusion protein (MalE-*Eco*MinC_NTD_) was reported to form oligomers (a higher order than the dimer) (Hu & Lutkenhaus, 2000 ▶). As we observed in the crystal structure, the swapping of the N-terminal β strands are critical for the dimer formation. Thus the N-terminal fusion in MalE- *Eco*MinC_NTD_ probably interferes with proper dimer formation. By using unfused-*Eco*MinC_NTD_, we observed that *Eco*MinC_NTD_ forms a stable dimer in solution and in the crystal, enabling us to conclude that *Eco*MinC dimerizes through both *Eco*MinC_CTD_ and *Eco*­MinC_NTD_. It is noteworthy that between *Eco*MinC_CTD_ and *Eco*MinC_NTD_ is a long linker (∼25 residues) that is not present in *Tma*MinC (Fig. 3 ▶). This long linker makes possible independent dimerization of *Eco*MinC_NTD_ and *Eco*MinC_CTD_. Consistent with that idea, we observed in a chemical crosslinking experiment that at a high *Eco*MinC concentration there was greater oligomer formation than could be explained through alternative dimer formation by *Eco*MinC_NTD_ and *Eco*MinC_CTD_ (data not shown).

### 3.2. Model for interaction between polymeric FtsZ and dimeric *Eco*MinC

**Figure 4:**
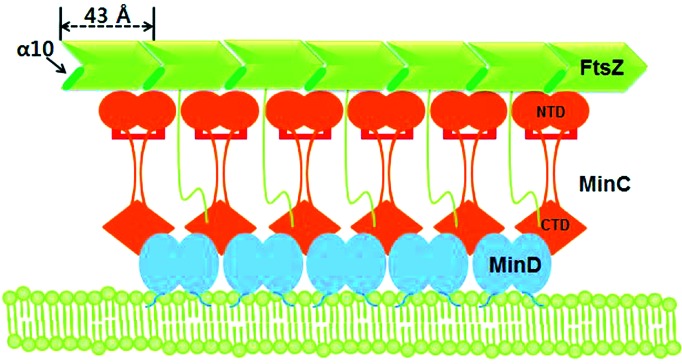
Schematic model of MinC binding to polymeric MinD and FtsZ. MinD is known to bind to the membrane *via* an extreme C-terminal amphipathic helix. MinC_CTD_, bound to MinD, interacts with the C-terminal tail of FtsZ. MinC_NTD_ binds to α10 of FtsZ located at the interface of FtsZ subunits within a FtsZ filament. The distance between α10s within the FtsZ polymer is about 43 Å. Dimensions of MinC_NTD_ are 40 Å × 52 Å (Fig. 2*a* ▶).

Polymeric FtsZ (Z ring) is located at the mid-site in cells undergoing normal cytokinesis. Underlying this process is the negative regulation of aberrant polymeric FtsZ by the MinC/D complex. MinD recruits MinC near the membrane through interaction with the conserved RSGQ sequence of MinC (Ramirez-Arcos *et al.*, 2004 ▶; Zhou & Lutkenhaus, 2005 ▶), which leads to the MinC dimer being situated between two dimeric MinD molecules (Wu *et al.*, 2011 ▶) (Fig. 4 ▶). It has also been reported that, upon formation of the MinC/D complex, MinC_NTD_ binds to α10 of FtsZ, which is located at the interface between FtsZ subunits (Shen & Lutkenhaus, 2010 ▶), while MinC_CTD_ binds to the C-terminal tail of FtsZ (Shen & Lutkenhaus, 2009 ▶) (Fig. 4 ▶). In that context, our present findings make it reasonable to suggest that dimeric *Eco*MinC_NTD_ binds to α10 of FtsZ, as the surface for FtsZ binding is located in the α-helical subdomain, and the C-terminus of *Eco*MinC_NTD_ is located in the β-sheet subdomain (Fig. 2*a* ▶). In addition, the dimensions of dimeric *Eco*MinC_NTD_ (40 × 52 Å; Fig. 2*a* ▶) match well with the repetition of α10 of FtsZ polymer (43 Å, Fig. 4 ▶).

Recently, Blasios *et al.* (2013 ▶) identified the binding sites for MinC in *Bacillus subtilis* FtsZ and found that they differ significantly from those in *E. coli*. They proposed that the mechanism of MinC action differs between these two species, being primarily at the level of inhibiting FtsZ filament bundle formation in *B. subtilis*. It is noteworthy that the N-terminal sequence of *B. subtilis* MinC (*Bsu*MinC), corresponding to the first β strand, aligns better with *Tma*MinC than with *Eco*MinC or *Sty*MinC (Fig. 3 ▶). We would therefore expect *Bsu*MinC_NTD_ to contain two short strands forming an antiparallel sheet similar to that in *Tma*MinC_NTD_, which would result in monomeric *Bsu*MinC_NTD_. This could explain why *Eco*MinC_NTD_ and *Bsu*MinC_NTD_ interact with different regions of FtsZ.

Taken together, the results of our structural study of *Eco*MinC_NTD_ reveal that domain swapped dimerization is a likely mode of interaction with polymeric FtsZ. To unravel the underlying mechanism of this interaction and physiological function of the domain swapping in the *Eco*MinC_NTD_ dimer, additional biochemical and cell-based experiments are required, using the wild type and mutants, which stabilize monomeric *Eco*MinC_NTD_ in the aspect of FtsZ interaction and cell division inhibition. Furthermore, it will be intriguing to compare the differences in functional regulation by the domain-swapped dimers, such as *Eco*MinC, with other MinCs that do not have domain swapping, such as *Thermotoga maritima* (and, probably, *Bacillus subtilis*; Fig. 3 ▶).

## Supplementary Material

PDB reference: 4l1c

## Figures and Tables

**Table 1:** Data collection and refinement statistics for *E. coli* MinC_NTD_ Values in parentheses are for the highest-resolution shell.

Data collection	
X-ray source	PAL-4A
Wavelength (Å)	1.0000
Space group	*P*2_1_2_1_2_1_
Unit-cell dimensions (Å)	*a* = 52.7, *b* = 54.0, *c* = 64.7
Resolution (Å)	50–2.30 (2.34–2.30)
Observed reflections	99925
Unique reflections	8799
Multiplicity	11.3 (10.2)
Completeness (%)	99.8 (98.8)
*R*_merge_ † (%)	6.5 (41.5)
*I*/σ(*I*)	13.8 (4.6)

Refinement statistics	
Resolution (Å)	27.8–2.3
*R*_work_ ‡/*R*_free_ § (%)	0.229/0.263
R.m.s.d bond length (Å)	0.014
R.m.s.d bond angle (°)	1.418
Ramachandran	
Favored (%)	95.8
Allowed (%)	3.6
Outliers (%)	0.6
PDB id	4l1c

† *R*_merge_ = ∑_*hkl*_∑_*i*_|*I*_*i*_(*hkl*) − 〈*I*(*hkl*)〉|/∑_*hkl*_∑_*i*_ *I*_*i*_(*hkl*), where *I*_*i*_(*hkl*) is the intensity of the *i*th observation of reflection *hkl* and 〈*I*(*hkl*)〉 is the average intensity of reflection *hkl*.

‡ *R*_work_ = Σ||*F*_o_| − |*F*_c_||/Σ|*F*_o_|.

§ *R*_free_ calculated with 10% of all reflections excluded from refinement stages using high-resolution data.
